# Adult-Onset Leukoencephalopathy With Axonal Spheroids and Pigmented Glia: Review of Clinical Manifestations as Foundations for Therapeutic Development

**DOI:** 10.3389/fneur.2021.788168

**Published:** 2022-02-03

**Authors:** Spyros Papapetropoulos, Angela Pontius, Elizabeth Finger, Virginija Karrenbauer, David S. Lynch, Matthew Brennan, Samantha Zappia, Wolfgang Koehler, Ludger Schoels, Stefanie N. Hayer, Takuya Konno, Takeshi Ikeuchi, Troy Lund, Jennifer Orthmann-Murphy, Florian Eichler, Zbigniew K. Wszolek

**Affiliations:** ^1^Vigil Neuroscience, Inc, Cambridge, MA, United States; ^2^Massachusetts General Hospital, Boston, MA, United States; ^3^Clinical Neurological Sciences, Western University, London, ON, Canada; ^4^Neurology Medical Unit, Karolinska University Hospital, Stockholm, Sweden; ^5^Department of Clinical Neuroscience, Karolinska Institute, Stockholm, Sweden; ^6^National Hospital for Neurology and Neurosurgery, London, United Kingdom; ^7^University of Leipzig Medical Center, Leipzig, Germany; ^8^Department of Neurodegenerative Diseases, Hertie-Institute for Clinical Brain Research and Center of Neurology, University Hospital Tuebingen, Tuebingen, Germany; ^9^German Research Center for Neurodegenerative Diseases, Tuebingen, Germany; ^10^Brain Research Institute, Niigata University, Niigata, Japan; ^11^Department of Pediatrics, University of Minnesota, Minneapolis, MN, United States; ^12^Department of Neurology, University of Pennsylvania, Philadelphia, PA, United States; ^13^Massachusetts General Hospital, Boston, MA, United States; ^14^Mayo Clinic, Jacksonville, FL, United States

**Keywords:** adult-onset, leukoencephalopathy, leukodystrophy, axonal spheroids, pigmented glia, HDLS, ALSP, *CSF1R*

## Abstract

A comprehensive review of published literature was conducted to elucidate the genetics, neuropathology, imaging findings, prevalence, clinical course, diagnosis/clinical evaluation, potential biomarkers, and current and proposed treatments for adult-onset leukoencephalopathy with axonal spheroids and pigmented glia (ALSP), a rare, debilitating, and life-threatening neurodegenerative disorder for which disease-modifying therapies are not currently available. Details on potential efficacy endpoints for future interventional clinical trials in patients with ALSP and data related to the burden of the disease on patients and caregivers were also reviewed. The information in this position paper lays a foundation to establish an effective clinical rationale and address the clinical gaps for creation of a robust strategy to develop therapeutic agents for ALSP, as well as design future clinical trials, that have clinically meaningful and convergent endpoints.

## Introduction

Adult-onset leukoencephalopathy with axonal spheroids and pigmented glia (ALSP) is a rare neurologic disorder that is characterized by demyelination of white matter of the brain, swollen axons and pigmented glial cells. The term ALSP encompasses two clinicopathologically similar entities that were previously known as hereditary diffuse leukoencephalopathy with spheroids (HDLS) and pigmentary orthochromatic leukodystrophy (POLD) (1). POLD was first described in 1936 in a family with adult-onset leukodystrophy (2). The term HDLS was first coined in 1984 to describe a Swedish family with adult-onset leukoencephalopathy in which axonal dilatations (spheroids) were a prominent feature (3). However, the original Swedish family (HDLS-S) was recently found to carry a different genetic makeup with the affected family members displaying the alanyl-transfer (t) RNA synthetase (*AARS*) gene mutation as the likely cause of Swedish type HDLS with spheroids (4). Thus, this family belongs to yet another class of genetic disorders identified as *AARS*-related leukoencephalopathy (5–7).

Several cases of POLD that fulfill all criteria for HDLS prior to the discovery of the *AARS* gene mutation causing HDLS in the Swedish family, except heritability, have been described in the literature. These cases are likely sporadic due to *de novo* mutations or issues of reduced disease penetrance (8, 9). Identification of common mutations in the kinase domain of *CSF1R*, a gene that regulates mononuclear cell lineages, including microglia in both HDLS and POLD (1), has provided additional evidence that HDLS and POLD should be regarded as a single disease entity (10, 11). In line with this, the diseases are now summarized as *CSF1R*-related leukoencephalopathy and for the purposes of this review are collectively referred to as ALSP.

The genetic, structural and neuropathophysiologic abnormalities of ALSP result in multiple neurologic symptoms, such as cognitive dysfunction, movement disorders, motor impairment, familial dementia and subcortical gliosis of the Neumann type and neuropsychiatric complications, that result in diminishing quality of life and eventual premature death (12–17). At present, some symptoms of ALSP are treated off-label with existing US Food and Drug Administration (FDA)-approved drugs that elicit variable levels of short-term efficacy. However, these symptomatic therapies have limited efficacy and do not target the etiology or the most debilitating symptoms, such as rapidly progressive cognitive impairment of ALSP (14). Future novel therapies that focus on the neuropathophysiologic features that underlie ALSP are essential to adequately reverse, delay or stop progression and improve quality of life in patients who are afflicted with this incapacitating disorder.

This review of the published literature was conducted to critically evaluate the clinical characteristics of ALSP as a foundation for rigorous strategy to develop therapeutic agents targeting ALSP. The review highlights key considerations for design of human clinical trials for ALSP including clinically meaningful and convergent endpoints that will lead to the development of safe and effective therapies for this orphan neurologic disorder.

## Literature Search Strategy

Published data for the clinical characterization of ALSP were limited by the small number of patients who are afflicted with this adult-onset leukoencephalopathy. Observational case studies comprised the majority of publications for ALSP. The primary literature search for published and in-press clinical studies of ALSP were obtained from a MEDLINE search in the timeframe of January 1, 1980 through October 31, 2020. ALSP literature subsequent to October 31, 2020 was also monitored through MEDLINE. Literature relevant to proposed efficacy endpoints, future interventional clinical trials of ALSP and burden of care due to unmet medical needs was derived from MEDLINE and PUBMED during the above time periods. Review of all publications was restricted to articles in English or translated into English. The following primary search terms were utilized to identify ALSP-related publications:

- Adult-onset leukodystrophy with neuroaxonal spheroids and pigmented glia
- Adult-onset leukoencephalopathy with axonal spheroids and pigmented glia
- ALSP
- *CSF1R*-related leukoencephalopathy
- Hereditary diffuse leukoencephalopathy with spheroids
- HDLS
- Pigmentary type of orthochromatic leukodystrophy
- POLD.

## Genetics

ALSP is primarily inherited as an autosomal dominant disorder with *CSF1R* gene mutations as the most common mutation to date (14, 18, 19). At least 106 different *CSF1R* mutations have been identified in approximately 300 cases published in the peer-reviewed literature worldwide (6, 17–32). There is no major correlation of genotype and phenotypes. Family members with identical *CSF1R* gene mutations do not share the same clinical phenotype (6, 14). However, patients harboring truncating mutations of *CSF1R* as well as those that lead to nonsense-mediated decay have been shown to have an earlier age of onset than ALSP patients with substitution mutations (32). Although *de novo* mutations of *CSF1R* have been reported, they are less common (9, 20). Penetrance of ALSP associated with *CSF1R* mutations is high, but incomplete, due to *de novo* mutations and genetic mosaicism (6, 9, 13, 33).

**Figure 1:**
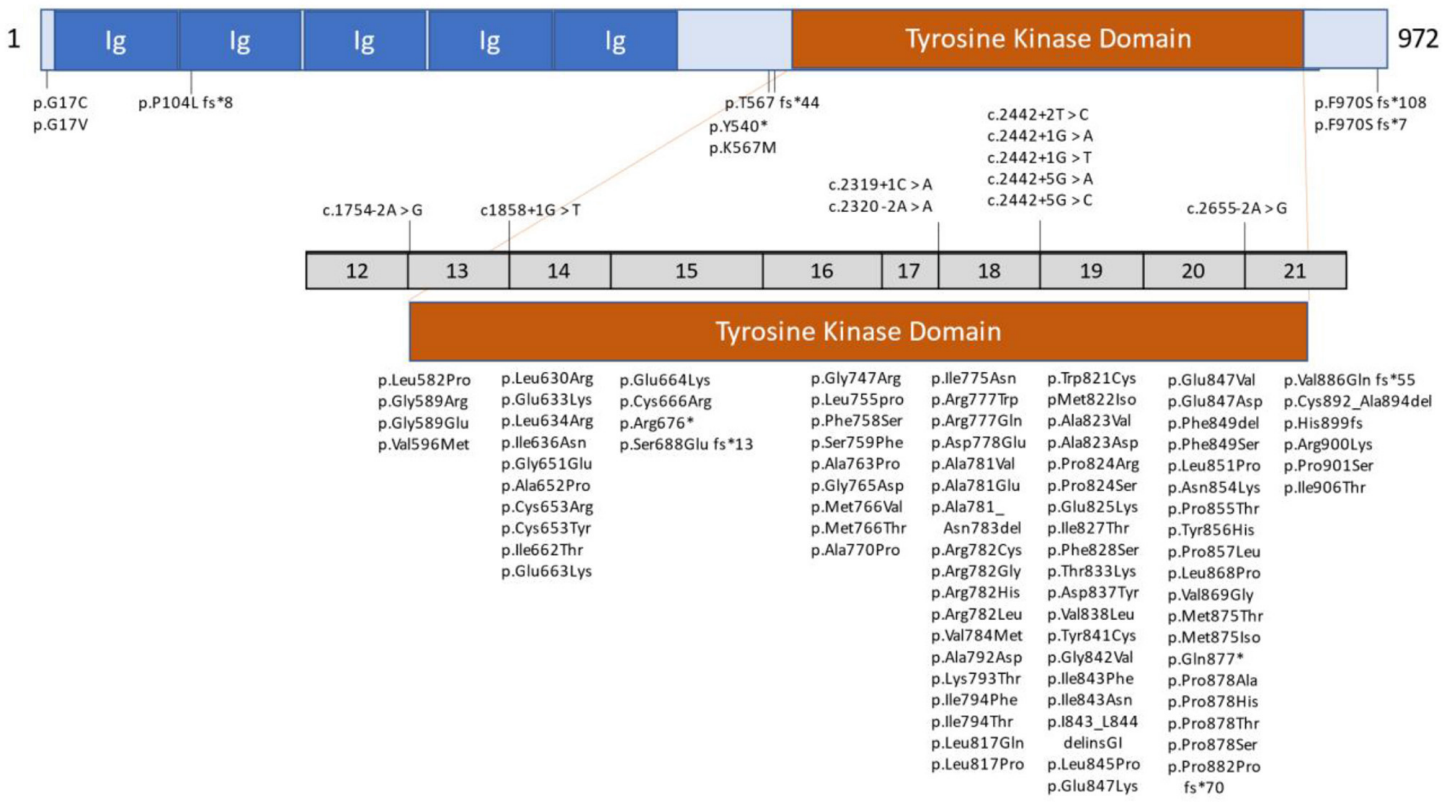
Schematic graph of the *CSF1R* protein and overview of *CSF1R* mutations identified in patients with *CSF1R*-related leukoencephalopathy. *CSF1R*, colony-stimulating factor 1 receptor; Ig, immunoglobulin domain. Revised/updated version reprinted with permission from (31).

The non-mutated *CSF1R* gene consists of 22 exons. Its normal gene product is the CSF1R protein, a cell-surface transmembrane tyrosine kinase receptor with two known ligands for cytokine colony-stimulating factor 1 (CSF1) and IL-34. Most mutations of *CSF1R* that are associated with ALSP are located in the tyrosine kinase domain (TKD), most frequently in exons 18 and 19 (6). However, novel *CSF1R* variants in regions such as the signal peptide, immunoglobulin (Ig) domain, transmembrane domain, other exons of the TKD, and at the C-terminus, have been reported (6, 18, 21, 23, 31). Additionally, unique splice site variants, deletion/insertion mutations and frameshift mutations have been reported in introns and exons that span nearly all of *CSF1R* (6, 17, 19, 21, 23, 24, 26, 31). Figure 1 illustrates the location of *CSF1R* mutations in patients with *CSF1R*-related leukoencephalopathy. A more detailed presentation of genetic mutations in *CSF1R*-related leukoencephalopathy is provided in Supplementary Table 1.

Survival, development, proliferation, and activation of mononuclear phagocytic cells and central nervous system (CNS) microglia are regulated by *CSF1R*. *CSF1R* gene mutations are considered an underlying cause of primary brain microgliopathies and are linked to CNS damage of ALSP (20, 34–36).

Biallelic mutations in the *AARS* gene have been detected in approximately 20 patients with late-onset leukoencephalopathy who did not have a *CSF1R* mutation (37). The *AARS2* gene encodes a mitochondrial enzyme that is essential for loading alanine onto tRNA during mitochondrial translation (37). Most of these patients displayed an autosomal recessive inheritance of *AARS2*-related leukoencephalopathy with symptoms and brain neuropathology similar to ALSP (37–40).

Both *CSF1R*- and *AARS2*-related leukoencephalopathy share several neurological symptoms and can present with similar white matter involvement, predominantly in the frontoparietal and periventricular regions. However, differences in radiologic images between patients with *CSF1R* and *AARS2* gene encoding mutations have been reported in the corpus callosum, in regions with severe brain atrophy and in patients with *AARS2* gene mutations lacking the unique ALSP-associated calcifications that are seen on computed tomography (CT) (13, 37, 41). Unlike *CSF1R*, the *AARS2*-related phenotype has not been restricted to adults, with some cases reported during adolescence (42). The changes in nomenclature for leukoencephalopathies are a result of additional understanding of pathology and genetics. The five main classifications of leukoencephalopathies are *CSF1R*-related leukoencephalopathy, *AARS2*-related leukoencephalopathy, *AARS1*-related leukoencephalopathy, HDLS-S-related leukoencephalopathy and *CSF1R/AARS1/AARS2*-negative ALSP. The last classification represents cases with a neuropathological diagnosis of ALSP without mutations in the *CSF1R* and *AARS2* genes.

Further analyses of brain images and modern neuropathology are necessary to definitively characterize *AARS2*-related leukoencephalopathy. However, due to limited and conflicting data associated with *AARS2*, as well as no known molecular signaling convergence between *CSF1R* and *AARS2*, this clinical review has focused on the term ALSP, a *CSF1R*-related leukoencephalopathy.

## Neuropathology

**Figure 2:**
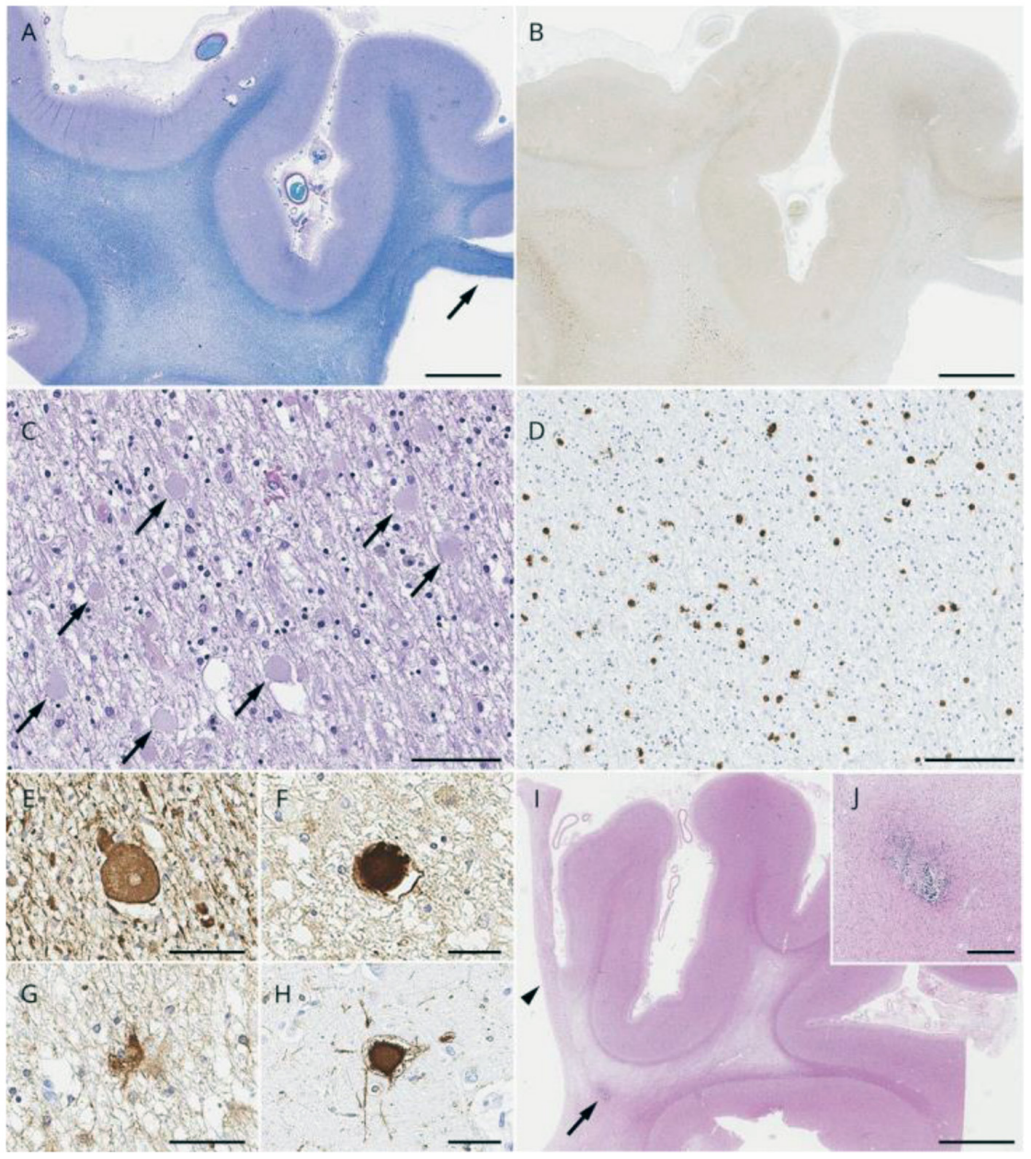
Pathologic light microscopic findings from cases with Colony-Stimulating Factor 1 receptor (*CSF1R*)-related leukoencephalopathy. **(A–H)** A 78-year-old man with *CSF1R* p.M875T. At 71 years of age, he developed cognitive impairment followed by personality and behavior change, depression, executive dysfunction, apraxia, parkinsonism, and pyramidal weakness. He died after 7 years of disease duration. **(A)** Luxol fast blue stain shows severe myelinated fiber loss in the superior frontal and cingulate white matter, whereas the U-fibers are relatively spared. Note the thinning of the corpus callosum (arrow). **(B)** The axonal spheroids in the affected white matter are stained with amyloid precursor protein (APP). **(C)** Numerous axonal spheroids (arrows) are seen within the frontal white matter (hematoxylin and eosin). **(D)** 68-immunopositive macrophages in the frontal white matter. **(E,F)** An axonal spheroid in the white matter depicted by phosphorylated neurofilament (SMI31) **(E)** and APP **(F)**. **(G)** A bizarre astrocyte in the white matter (αB-crystallin). **(H)** A ballooned neuron in the superior frontal cortex (αB-crystallin). **(I,J)** A 55-year-old woman with autopsy-confirmed adult-onset leukodystrophy with neuroaxonal spheroids and pigmented glia, but genetic testing was not performed because DNA was unavailable. **(I)** Note the small, calcified lesion (arrow) located in the pericallosal region. An arrowhead indicates the paper-like atrophy of the corpus callosum. **(J)** An enlarged image of the calcification. Bars in A, B, and I = 5 mm; C and D = 100 μm; E, F, G, and H = 50 μm; and J = 400 μm. Reprinted with permission from (6).

Histopathologic evaluation (light and electron microscopy) of brain tissue from biopsies and autopsies of patients with ALSP shows multiple morphologic alterations (6, 14, 17, 29, 43–46). One of the principal neuropathologies consists of vacuolated and demyelinated white matter that is found primarily within the corpus callosum, pyramidal tracts and periventricular region of the frontal and parietal lobes. The degenerate white matter is often associated with deteriorating neurons and axonal spheroids that contain neurofilaments, amyloid and ubiquitin. The axonal pathology is accompanied by macrophages that are engorged with lipid and myelin. Other characteristic neuropathological findings of ALSP include deformed astrocytes and pigmented (iron or lipofuscin) microglia cells that decrease in function and number with progression of the disorder. Representative lesions from cases of ALSP are shown in Figure 2.

ALSP is associated with leaky blood brain barrier (BBB) and cerebrovascular abnormalities similar to cerebral amyloid angiopathy (CAA) (47). Post-mortem brain sections from ALSP patients were stained for Claudin-5, a key mediator of tight junction function at the BBB in areas of dense amyloid-beta integrity and demonstrated a non-linear distribution of Celaudin-5 in tandem with extravasation of IgG and fibrinogen. These findings implicate BBB disruption. Perivascular localization of CD68- and CD163-positive cells in the brain of ALSP patients suggest that peripheral macrophages are recruited to the vasculature (47). Identification of donor chimerism in the CSF (cerebrospinal fluid) obtained from patients who underwent hematopoietic stem cell transplantation (HSCT) further supports this observation (48).

Additionally, the proinflammatory cytokine, granulocyte macrophage colony stimulating factor (GM-CSF), was shown to be significantly upregulated in the gray matter on post-mortem analysis of brain tissue from patients with ALSP and migration and adhesion-related molecules derived peripheral blood monocytes were also significantly upregulated, suggesting widespread immune dysfunction (28, 32). More robust characterization of the innate and adaptive immune system throughout the course of disease progression is warranted to understand its potential role in ALSP pathobiology.

## Imaging

**Figure 3:**
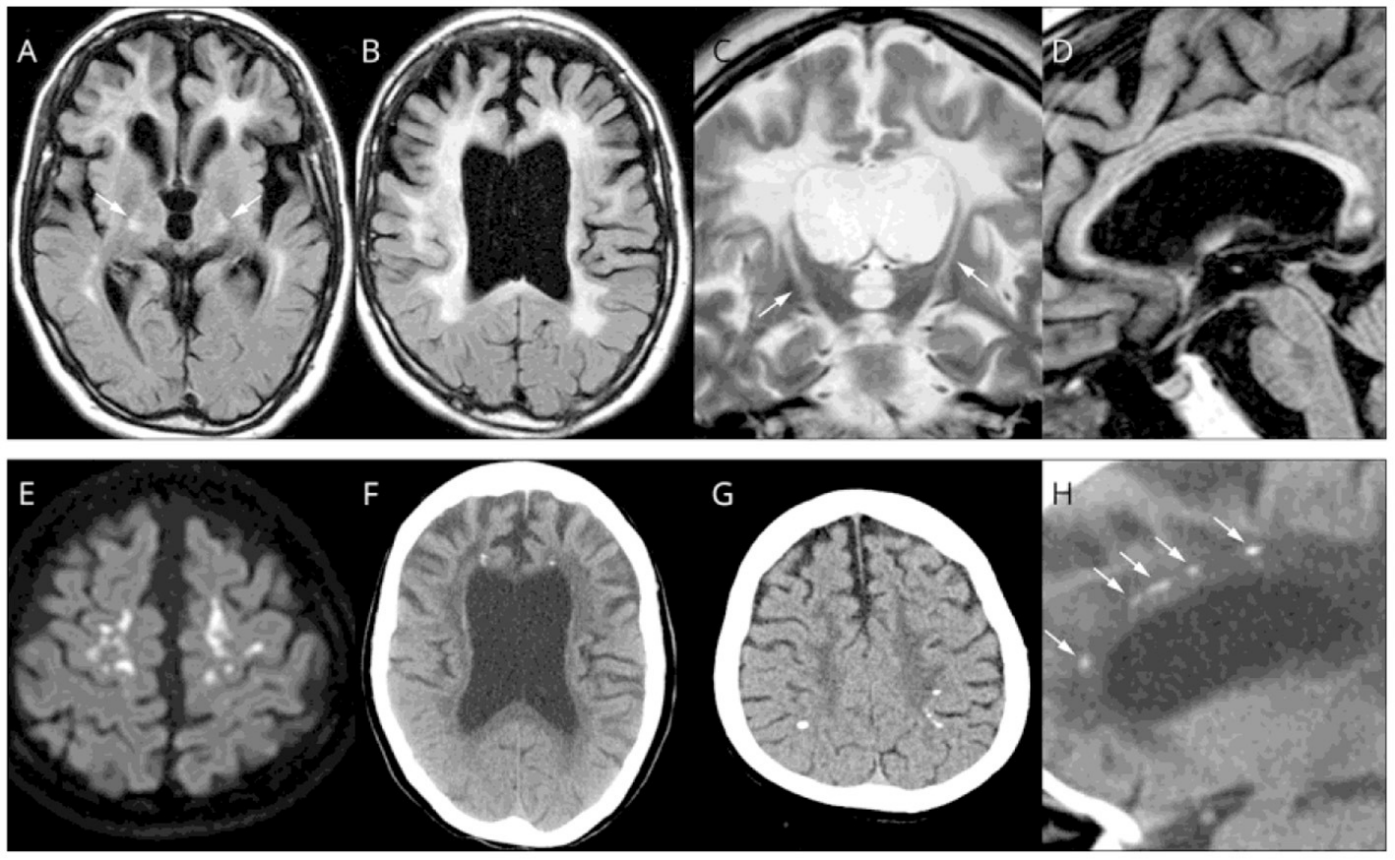
Brain magnetic resonance imaging (MRI)/computed tomography (CT) findings from cases of Colony-Stimulating Factor-1 Receptor (*CSF1R*)-related leukoencephalopathy. **(A–D,F,H)** A 44-year-old woman with *CSF1R* p.G589R. **(E)** A 27-year-old woman with *CSF1R* c.2442 + 5 G > A. **(G)** A 31-year-old woman with *CSF1R p.A652P*. **(A,B)**, Bilateral diffuse white matter hyperintensity with pyramidal tract involvement (arrows in **A**), cortical atrophy, and enlarged lateral ventricles on fluid-attenuated inversion recovery MRI. **(C)** Longitudinal pyramidal tract involvement (arrows) on coronal T2-weighted image. **(D)** Thinning of the corpus callosum with hyperintensity on sagittal fluid-attenuated inversion recovery image. **(E)** Hyperintensity lesions in the subcortical white matter on diffusion-weighted image. **(F)** Small calcifications located bilaterally near the anterior horns of the lateral ventricles on brain CT image. **(G)** Calcifications in parietal subcortical white matter. **(H)** Stepping-stone appearance of calcifications (arrows) in the frontal pericallosal region on sagittal CT image. Reprinted with permission from (6).

Magnetic resonance imaging (MRI) of patients with ALSP typically demonstrates abnormal white matter signal with hyperintense (T2) and fluid-attenuated inversion recovery (FLAIR) lesions and diffusion tensor imaging (DTI) identifies diffusion restriction lesions (6, 13, 49–52). Figure 3 presents the typical abnormal brain MRI findings in patients with ALSP.

White matter lesions are some of the most common neuroradiological findings on MRI in ALSP. They can be symmetric or asymmetric, patchy or confluent and tend to involve different lobes of the brain during the evolution of the disease. A study of 122 patients with ALSP and *CSF1R* mutations reported the presence of bilateral white matter lesions in 96% of the patients (13). Similarly, a subsequent MRI study found bilateral, predominantly frontal and parietal, T2/FLAIR white matter hyperintensities associated with T1 hypointensities in 16 patients with ALSP (22). This study also reported progression of white matter lesions on imaging in a subset of 13 patients with follow-up MRIs. White matter lesions were identified with either a patchy or confluent appearance and the occipital and temporal lobes showed white matter lesion involvement in later stages of the disease (51). A summary review of white matter lesions confirmed that they are often asymmetric, patchy, and focal especially in the early stages of the disease, but with time they become confluent (50). Lesions are found predominantly in frontoparietal and periventricular areas. Overall, previously published MRI data identify some distinctive features of white matter lesions in patients with ALSP. These data are included in the diagnostic criteria (53) that may prove useful in assessing disease progression.

Focal and global brain atrophy as well as thinning of cortex and corpus callosum are other hallmarks found in ALSP. Brain atrophy was shown in 94% of cases (15/16), predominant in the frontal (40%, 6/15) or frontoparietal (53%, 8/15) areas, and progressed in association with larger white matter lesions over time (22). Dilation of the lateral ventricles was identified in a population of 122 subjects (13). Thinning of the corpus callosum was evident in 88% (23/26) and cortical atrophy in 92% (24/26) of patients (13). Similarly, a case study revealed corpus callosum abnormalities were present in 81% (13/16) and atrophy in 88% (14/16) (22). A striking finding in ALSP in some patients is areas of diffusion restriction that can be confused with stroke (often leading to misdiagnosis), but do not occur in vascular distributions. In summary, MRI data across several studies consistently demonstrates brain volumetric changes including cortical and corpus callosum thinning in ALSP patients highlighting the importance of utilizing advanced analysis techniques for systematic investigation of regional brain volume and cortical thinning as imaging markers for progression of ALSP.

Overall, MRI, as a non-invasive approach, has consistently shown a variety of features of ALSP that demonstrate a strong radiological, pathological and clinical correlation. Four stages of ALSP have been described on the basis of degree of axon loss (54). Stage I is depicted as patchy axon decline in cerebral white matter without atrophy. Stage II reveals large patchy axon loss with slight atrophy of cerebral white matter and slight dilation of the lateral ventricles. Stage III presents wide-spread axon loss in cerebral white matter and dilation of the lateral and third ventricles without prominent axon loss in the brainstem and cerebellum. Stage IV is characterized by extensive damage of cerebral white matter with pronounced dilation of the ventricles and loss of axons in the brainstem and/or cerebellum. With MRI, patients with ALSP have been shown to exhibit bilateral asymmetric white matter lesions that were patchy (as described in histopathology of Stage I), particularly within frontal and parietal lobes in early stages of the disorder. These lesions become confluent and expand into temporal and occipital lobes in later stages of the disease (6, 13, 22, 50, 51), as shown in histopathology of ALSP during Stages III, IV. Based on clinical manifestations of the disease, a frontal lobe syndrome is typical in the early course of the disease and aligns with the histopathology and imaging findings. The thinning of corpus callosum is evident from histopathology of ALSP Stage I and is also evident in the early phases of the disease with MRI. Similarly, both histopathology and MRI indicate that ventricular dilation begins in relatively earlier stages of the disease with consistent progression. Consistent with histopathology, brain atrophy starts in the cerebrum and can affect other brain regions over time (22). Other common findings of histopathology and MRI includes thinning of cerebral cortex or cortical atrophy as well as degeneration of projection fibers (e.g., corticospinal tracts, internal capsule, pyramidal tracts). Notably, some features such as involvement of deep gray matter are rarely seen on MRI and are only present in histopathology of Stage IV. Contrast-enhanced MRI studies in genetically diagnosed ALSP have also revealed a breakdown of the BBB integrity as indicated by the perilesional accumulation of the contrast agent gadobenate-disodium (Gd-BOPTA) (47).

**Figure 4:**
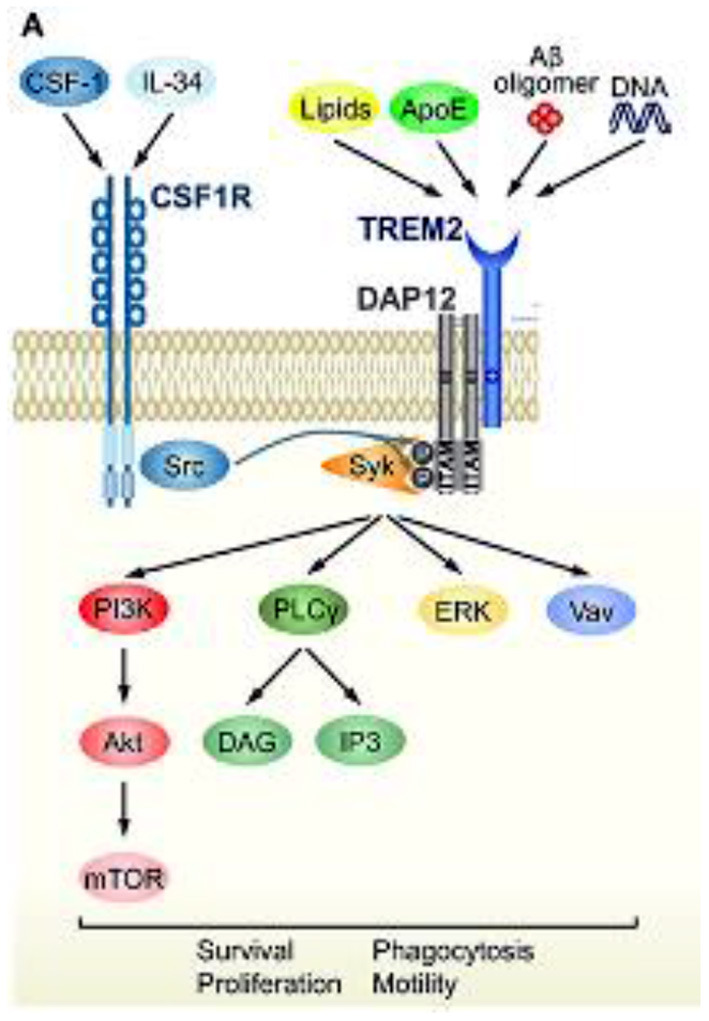
TREM2 signaling cascade and convergence with CSF1R signaling. Schematic representation of TREM2/DAP12 signaling in microglia. Ligands and downstream signaling of TREM2/DAP12. Of the known TREM2 ligands, only ligands that highly correlate with neural diseases are shown. Upon ligand binding to TREM2, two tyrosine residues within the ITAM motif of DAP12 are phosphorylated, which recruits Syk kinase to activate downstream signaling molecules, such as ERK, PI3K, PLCγ, and Vav. Src, the main effector of CSF1R, is a kinase supposed to phosphorylate the ITAM tyrosine residues. Reprinted with permission form (94).

CT images have identified characteristic stepping-stone calcification of periventricular white matter near the frontal horns in up to 50% of patients with ALSP (6, 13) (Figure 4). Brain calcifications have been detected in patients with asymptomatic ALSP and the *CSF1R* mutation (13, 55). The relationship between formation of calcifications and the underlying disease mechanisms are unclear.

To date, alternate and advanced imaging techniques have been tested or proposed for use in ALSP and other leukodystrophies. For example, DTI, which quantifies the white matter integrity through analysis of water diffusivity, is a unique analysis technique applied to Diffusion Weighted Imaging (DWI) scans to characterize global diffusivity but also along specific directionality. A DTI study (56) was conducted in patients with leukodystrophy across different age groups and showed that DTI could be used to quantitatively capture changes in the white matter integrity in terms of magnitude and directionality and correlate the diffusivity abnormalities with ALSP progression, both spatially and temporally.

Magnetic resonance spectroscopy reveals markedly increased levels of cholin, myo-inositol and lactate and a decreased N-acetylaspartate peak in ALSP. These findings are clearly different from cerebral autosomal dominant arteriopathy with subcortical infarcts and leukoencephalopathy (CADASIL) (51).

Single photon emission computed tomography (SPECT) in combination with DaTscan^TM^ I-123 ioflupane injection and positron emission computed tomography (PET) have been used for brain imaging of neurodegenerative disorders such as ALSP, Alzheimer's Disease (AD) and Parkinson's Disease (PD). These imaging techniques provide valuable information on clinical diagnosis, pathology, disease progression and patient care (57). For example, SPECT with DaTscan^TM^ reveals loss of presynaptic dopamine transporter function in the striatum and ^18^F-[fluorodeoxyglucose] PET illustrates diffuse cortical hypometabolism primarily in fronto-parietal areas of patients with ALSP (1, 6, 58). Finally, optical coherence tomographic imaging has also shown atrophy of the retinal nerve consistent with optic tract degeneration in a single case of ALSP (59).

## Prevalence

Mendelian adult-onset leukodystrophies are a spectrum of rare, chronic, complex and progressive neurologic disorders that affect the white matter of the CNS, with a total estimated global prevalence of 300 cases per million (60). However, dedicated epidemiological studies are missing to support the available prevalence data. The worldwide incidence of these disorders has more recently been reported to be 5 per 100,000 (61). Future studies are critical to fully evaluate the epidemiology of adult-onset leukodystrophies.

Historically, the diagnosis of ALSP was previously dependent on histopathologic findings from brain biopsies or autopsies. The number of patients who were definitively diagnosed with ALSP was extremely limited until the discovery of *CSF1R*-related gene mutations. Although still rare, genetically diagnosed *CSF1R*-related ALSP has been increasingly recognized around the world (identified in Canada, China, Croatia, Germany, Italy, Japan, Netherlands, Norway, Poland, Saudi Arabia, Sweden, South Korea, Taiwan, United Kingdom, and United States) since 2012. Therefore, this disease obviously has global distribution and many patients may still be underdiagnosed (6).

In a series of leukodystrophy cases, 12 probands or cases with mutations in the *CSF1R* gene were identified (58). These mutations were responsible for 11% (12 of 114) of the overall series, including 7% (6 of 88) of the clinical series of patients with leukodystrophy on brain MRI and 20% (5 of 25) of the patients with a histopathological diagnosis of HDLS. In a smaller series in which 25 patients with adult-onset leukoencephalopathy of unknown cause were screened for mutations in the *CSF1R* gene, six patients (24%) showed a *CSF1R* mutation (9).

One study estimated the frequency of *CSF1R*-related leukoencephalopathy at 10% (5 of 48) of adult-onset leukoencephalopathies and suggested that it could be the most common type (5). Approximately one-third of patients with genetically proven *CSF1R*-related ALSP have been reported in the adult-onset leukoencephalopathy population in Japan (13). Based on an increasing number of such case report studies, patients with *CSF1R*-related ALSP constitute an overall global incidence range of 10-25% of the adult-onset leukodystrophy population (14).

As of 20 May 2021, based upon the available peer-reviewed literature, the total estimated global prevalence of mendelian adult-onset leukodystrophies is 300 cases/million and 10-25% of those cases are projected to be *CSF1R*-related ALSP patients. In the United States population, the estimated prevalence of *CSF1R*-related ALSP patients is 9,970-24,926 (60). Additional epidemiological studies are warranted to fully characterize the prevalence of ALSP.

## Clinical Course

The mean age for onset of symptoms of ALSP is primarily in the 4th decade but can span from early adulthood to the 8th decade (14). The cumulative incidence of *CSF1R*-associated ALSP increases from 10% at 27 years of age, to 50% at 43 years of age and to 95% at 60 years of age, for a median age of 43 years (13). One of the few large case studies and literature reviews (122 patients) reported that the onset of ALSP symptoms occurs on average earlier in women (40 years) than in men (47 years) (13). Progression of the disorder from onset of symptoms to death varies from 2 to >30 years (mean, 6-8 years) (13, 62).

ALSP is clinically delineated by cognitive dysfunction with neuropsychiatric and motor symptoms. Signs and symptoms tend to be non-specific in the early stages of the disorder and may be difficult to distinguish from other neurological disorders in the absence of genetic confirmation of *CSF1R* gene mutations. The presenting cognitive, neuropsychiatric and motor symptoms and the rate of progression of symptoms varies among patients and within family members who carry the same *CSF1R* mutation (14). A frontal lobe syndrome, which is characterized by cognitive impairment, neuropsychiatric changes (depression, minimal social inhibition and poor insight) and limited motor dysfunction, is usually detected during the early stages of ALSP, and some patients may exhibit mild seizures (14, 53, 63).

The type and incidence of initial core clinical symptoms of ALSP were rigorously evaluated in a case series and literature review of 106 out of 122 male and female patients diagnosed with ALSP (13). Cognitive impairment (59%), neuropsychiatric symptoms (44%) including anxiety, depression, apathy, indifference, abulia, irritability, disinhibition and distraction, motor dysfunction (38%) involving parkinsonian symptoms, gait disturbances and spasticity, speech difficulty (19%) and other symptoms (8%) including stroke-like episodes, sensory dysfunction, dizziness, fatigue and epilepsy, were reported in the early stages of ALSP. Approximately half of the 106 cases presented with 2 or more symptoms during early onset of ALSP. The rate of motor dysfunction was higher than the rate of cognitive impairment in women aged 20-29 years.

Progression of the neuropsychiatric aspects of ALSP can lead to further cognitive decline, severe depression, apathy, anxiety, irritability and familial dementia and subcortical gliosis of the Neumann type. Progressive motor symptoms are numerous and often include parkinsonian signs, such as tremor, rigidity, bradykinesia and postural instability. Additional progressive symptoms involve higher cortical functions (aphasia, agraphia, acalculia and very frequently apraxia), pyramidal detriments (hyperreflexia, hypertonia, spasticity and bilateral Babinski signs), bulbar signs (dysarthria, dysphagia and slurred speech), cerebellar abnormalities (ataxia, dysmetria, intention tremor and gait disturbances), and seizures. Development of sensory symptoms involve a diminished sense of vibration, position, touch and pain perception (6, 13, 14).

Rare progressive events of ALSP may include stroke-like episodes, bone cysts and optic and peripheral nerve dysfunction (6, 13, 14). Progression of the cognitive and motor deficits elicits a significant diminution in quality of life and disruption of employment. In the final stages of the disorder, loss of speech and voluntary movements, confinement to bed and a vegetative state are evident. Infections such as pneumonia often result in death (14).

## Diagnosis and Clinical Evaluation

The diagnosis of ALSP requires exclusion of autosomal dominant disorders with symptoms that overlap with ALSP, including Alexander disease (bulbar/pseudo bulbar signs, ataxia and spasticity), adult-onset autosomal dominant leukodystrophy (ADLD) (impaired cognition, pyramidal and cerebellar signs), cerebral autosomal dominant arteriopathy (subcortical infarcts and leukoencephalopathy, frontal lobe syndrome and white matter lesions), frontotemporal dementia (FTD) (frontal lobe pathology and pyramidal/extrapyramidal signs) and early-onset AD (executive dysfunction, personality derangement and similar onset of age) (14). Several other disorders such as autosomal recessive or X-linked leukodystrophies (vanishing white matter disease, metachromatic leukodystrophy, Krabbe's disease or X-linked adrenoleukodystrophy) as well as mitochondrial diseases (e.g., Leigh syndrome) have overlapping clinical symptoms with ALSP (14) and further complicate the differential diagnosis of ALSP, confirming the need for genetic testing, such as a leukodystrophy panel or whole exome testing (no family history) or gene testing (known family history).

Diagnosis of suspected ALSP is a multistep process (14, 30, 53, 64) that is typically initiated in patients, with a positive family history, who exhibit one or more of a variety of characteristic symptoms, such as personality changes, impaired cognition, memory derangement, bouts of depression or motor dysfunction, such as muscle weakness, impaired gait, slow movement, rigidity and tremor. MRI scans show distinctive radiological signs that are used to identify white matter lesions in the frontal region, corpus callosum and corticospinal tracts of the brain, as well as recognize enlarged ventricles due to cerebral atrophy.

A three-step MRI differential diagnostic approach has been proposed for several adult leukodystrophies, including ALSP. The first step involves identification of symmetric white matter. The second step consists of classification of the white matter into one or more of six patterns (parietal-occipital, frontal, periventricular, subcortical, brainstem involvement, and cerebellar involvement). The third step entails evaluation of five distinct characteristics (enhancement, lesions with signal intensity similar to CSF, susceptibility-weighted MRI signal intensity abnormalities, atypical peaks of MRI spectroscopy and spinal cord involvement) (65).

Medical history of family members can inform if ALSP is present in the lineage. The diagnosis of ALSP is verified through genetic testing and identification of a mutated *CSF1R* gene in most patients who are afflicted with ALSP (13, 14, 22, 30, 31, 53, 66).

Diagnostic criteria for *CSFIR*-related ALSP without a genetic test diagnosis have been developed and validated through a retrospective case study (Supplementary Table 2) (53), although a genetic test is recommended and finding of a known pathogenic *CSF1R* mutation supercedes these criteria. “Probable” and “possible” diagnostic designations were the outcomes based upon retrospectively specified clinical characteristics of ALSP, including age at onset ≤ 60 years, >2 impairments (e.g., cognitive, pyramidal, Parkinsonism or epilepsy), autosomal dominant inheritance and brain MRI/CT findings, such as bilateral cerebral white matter lesions and thinning of corpus callosum. Among the 83 patients that were positive for a *CSF1R* mutation, 50 (60%) had a “probable” diagnosis and 32 (39%) had a “possible” diagnosis of ALSP, resulting in a high level of sensitivity.

The specificity of an MRI diagnosis for *CSF1R*-related leukoencephalopathy was determined by retrospective evaluation of 53 cases of *CSF1R*-mutation-negative leukoencephalopathy and 32 cases of CADASIL. The MRI diagnostic algorithm excluded 22 cases (42%) that were negative for a *CSF1R* mutation and 28 cases (88%) that had a diagnosis of CADASIL. Moderate specificity for accurate diagnosis of *CSF1R*-related leukoencephalopathy relative to mutation-negative cases and the high specificity of *CSF1R*-related leukoencephalopathy relative to CADASIL cases were confirmed by this MRI diagnostic platform (53).

A more recent case study of 135 patients who were suspected of having adult-onset leukoencephalopathy due to *CSF1R* mutation was conducted to further evaluate the sensitivity and selectivity of diagnostic factors (30). As a result of genetic testing, 28 cases were positive for a *CSF1R* mutation and 107 cases were negative for a *CSF1R* mutation. Younger age at onset, Parkinsonian symptoms, reduction in the corpus callosum volume and presence of diffusion- restricted lesions were important predictors of *CSF1R*-positive cases whereas involuntary movements and brain stem or cerebellar atrophy were poor predictors of *CSF1R*-positive cases. The model confirmed high sensitivity for probable or possible *CSF1R*-related leukoencephalopathy at 81%, but like many models, suffered from lack of specificity at 14%.

Due to the above studies, the diagnostic accuracy for *CSF1R*-related leukoencephalopathy has significantly improved in recent years. After diagnosis of ALSP, clinical evaluations by a neurologist, psychiatrist, orthopedist, physical therapist and occupational therapist are periodically conducted for surveillance and for the treatment of symptoms with progression of the disorder. These assessments involve neurologic examination with cognitive and psychiatric evaluations, MRI of the brain for white matter lesions, brain atrophy and thinned corpus callosum and electroencephalograms (EEGs) for suspected seizures. Lumbar punctures have been proposed for the assessment of protein and cellular content. While not yet measured routinely for clinical management, serum NfL is an easily accessible, putative biomarker for following native disease course in ALSP (67) and potential treatment response.

## Current Treatment For Management of Symptoms

Currently, there are no regulatory-approved, disease modifying therapies for ALSP. Symptomatic treatments target the temporary relief of motor, mood and behavior symptoms and provide supportive care with the goal of maintaining quality of life as the disorder progresses (6). Although several regulatory-approved pharmacotherapies are prescribed off-label to treat the symptoms of ALSP, such as spasticity or seizures, that are comparable to those of other neurologic disorders (14), none of these pharmacotherapies target the cause or slow the progression of ALSP.

Dopaminergic drugs are used off-label to treat Parkinsonian symptoms with limited effectiveness because dopaminergic neurons are usually unaffected in the substantia nigra of patients with ALSP (68). As some symptoms of ALSP are similar to those of patients with AD, cholinesterase inhibitors have been prescribed but elicit minimal stabilization of symptoms of cognitive deficiency in patients with ALSP (69).

Antidepressants offer modest short-term efficacy for depression (14) and antipsychotic drugs are occasionally prescribed with caution for patients with ALSP because of the side effects including extrapyramidal symptoms and safety issues. There is risk of suicidal ideation associated with these agents but they may be useful in highly aggressive patients (14). Muscle relaxants for spasticity, anti-epileptic medications (e.g., benzodiazepines, gabapentin, valproic acid) generalized epileptic seizures and antibiotics for pneumonia and urinary tract infections have shown some benefit in patients with ALSP (6). Patients with ALSP generally fail to respond to immunomodulators, such as steroids, interferon and cyclophosphamide (6). The efficacy of other approaches, such as allogeneic HSCT (27, 48, 70, 71) and pre-symptomatic immunosuppression (72) have shown potential effectiveness in small retrospective case reports but have not yet been tested in controlled clinical trials.

Food consumption patterns and nutrition should be monitored throughout the disease course, as symptoms of ALSP can include dysphagia and gastrointestinal dysfunction, such as constipation and fecal incontinence. Urinary or urge incontinence may be alleviated by medication, scheduled toileting and intermittent or permanent catheterization of the urinary bladder. Physical, occupational and speech therapy are essential to maintain patient mobility, self-dependence and execution of daily living for as long as possible. Professional counseling is important to educate the patient and relatives on the symptoms and progression of ALSP and ensure a supportive family structure. However, due to the rareness of the disease, only a small number of experts in ALSP are available. Genetic counseling of the patient and relatives is necessary to explain the probability of inheritance and assist in the decision making of genetic testing of relatives (6, 14, 44). Referral to leukodystrophy communities/relevant foundations (e.g., Sisters' Hope Foundation) is essential for ongoing patient, family and caregiver support.

## Potential Neuropathophysiologic Biomarkers

A limited number of clinical case studies have been performed to evaluate the neuropathophysiologic biomarkers of ALSP. Abnormal levels of cytoskeletal proteins, such as NfL protein, tau protein, and glial fibrillary acid, were originally identified as potential biomarkers in 4 cases of ALSP (33, 62). In a more recent small study of 4 patients with *CSF1R*-mutation-positive ALSP, profiling of peripheral blood mononuclear cells (PBMCs) showed a basal proinflammatory phenotype (28).

NfL proteins support the cytoskeleton of neurons and myelinated axons, and elevated levels of NfL in CSF and blood are believed to be indicative of neuron death and axonal deterioration in a growing number of neurodegenerative disorders, including AD, PD, FTD, Lewy body dementia (LBD), progressive supranuclear palsy (PSP), Down syndrome, multiple sclerosis (MS), amyotrophic lateral sclerosis (ALS), Huntington's disease, X-linked adrenoleukodystrophy, spinocerebellar ataxia and Charcot-Marie-Tooth disease (73–85). A recent case control study demonstrated that serum and CSF levels of NfL protein were markedly higher in patients with symptomatic or presymptomatic (i.e., carriers) *CSF1R-*mutation-positive ALSP vs. that in healthy control subjects or in patients with MS (67). NfL protein levels in CSF were over 30-fold higher in patients with *CSF1R*-mutation-positive ALSP than in age-matched control subjects and serum NfL protein levels were significantly higher in symptomatic patients compared to presymptomatic *CSF1R* mutation carriers. Comparatively, patients with AD, ALS, FTD, LBD, and PSP have been shown to have CSF NfL levels approximately 2.3-fold, 7.2-fold, 3-fold, 2.8-fold, and 3.5-fold higher than healthy controls, respectively (82). Based on compelling evidence from case studies of ALSP and other neurodegenerative disorders, levels of NfL protein in serum and CSF may be predictive of clinical efficacy and should be rigorously tested as a potential biomarker and surrogate clinical endpoint for future clinical trials of therapeutic agents for ALSP.

Tau proteins regulate stability of microtubules in axons and neurons of the CNS. Abnormal levels of total tau protein concentrations (tauopathies) in CSF have been identified as a potential therapeutic biomarker in several neurodegenerative disorders, including AD, PD, progressive supranuclear palsy and ALSP (14, 86–88). Glial fibrillary acidic protein reinforces the cytoskeleton of astrocytes and increased expression often correlates with various types of neurodegenerative pathology. Increased concentrations of this protein have been observed in patients with ALSP and in patients with brain injuries (14, 89). Tau and glial fibrillary acidic proteins should also undergo further evaluation as potential biomarkers and surrogate endpoints for clinical trials of therapeutic agents for ALSP.

## Current and Proposed Interventional Clinical Studies of Therapeutics

Information on the use of HSCT for the treatment of ALSP is derived from four case studies with a limited number of patients (n=11) and inadequate controls. In the earliest case study, 1 of 4 siblings with ALSP underwent an allogeneic HSCT and experienced stabilization of ALSP symptoms and personality within 6 months, together with no progression in motor symptoms over the next 15 years (70). However, as the authors point out, the stabilization of symptoms in this transplanted patient may have developed spontaneously and therefore was potentially unrelated to HSCT. In a second case study of a single ALSP patient (71), the patient displayed deterioration with pyramidal symptoms and was confined to a wheelchair after 3 months of HSCT. Complications during HSCT involved hemorrhagic cystitis and pyelonephritis but both resolved after 6 months. Some stabilization of neurologic symptoms began at 6 months after allogeneic HSCT. Modest improvement in symptoms of ALSP occurred during the 30-month post-transplant period, including minimal limb mobility and stabilized cognition and mood on the Expanded Disability Status Scale (EDSS), concomitant with a decrease in lesions in the white matter through diffusion weighted imaging and reduced FLAIR hyperintensities on MRI. In a third case study of 2 patients with ALSP (27), some stabilization of cognitive deficits, functional status and gait impairment occurred over 2 years after transplant despite some continued neurological deterioration. There was no evidence of graft vs. host disease (GVHD), but a major infection developed in one patient and seizures were evident in the other patient. MRI scans of the brain demonstrated stabilization of FLAIR-related lesions in the white matter at 1 year, with continued stabilization over 2 years post-transplant. In a fourth case study, seven ALSP patients received HSCT at different stages of the disease (48) to determine the effect on progression. Six of the seven patients had several clinical evaluations at various time points post-transplant (one patient died post-transplant) and trended toward stabilization on motor examinations, cognitive scores and/or MIR abnormalities. Other minor clinical improvements tended to be empirical and subject to investigator bias because a negative control group was not included. Three patients developed GVHD. Overall, the four published case studies of HSCT with limited numbers of patients (*n* = 11) showed minimal to modest stabilization or improvement of ALSP symptoms with significant safety concerns including death of one patient.

Although the data on HSCT in ALSP are limited, given the challenges and risks of HSCT for the treatment of leukodystrophies and associated complications, clinical guidelines have been developed for pediatric patients with leukodystrophies and are likely applicable to adult patients as well (90). These guidelines focus on the assessment depending on the type of HSCT, patient eligibility, donor selection, conditioning regimen, pre-transplantation, supportive care and posttransplant follow-up. Early detection and therapy of leukodystrophies are highly recommended along with a close working relationship between families of patients and healthcare providers.

Microglial replacement, which represents a potential therapy for *CSF1R*-related leukoencephalopathy, has been the focal point of intense non-clinical research and may enter into clinical studies in the near future (91). Microglia are resident, innate immunity cells of CNS that monitor and maintain the robust physiology of the CNS. Microglia respond to various types of cellular and metabolic distress signals in the CNS and regulate responses for repair and remyelination of nerve fibers, phagocytosis of dead cells, maintenance of synapses and blood vessels and control of neuroinflammation associated with infection and carcinoma. The CSF1R cell-surface receptor is expressed predominantly on microglia in the brain (92) and in the spleen, placenta and appendix according to the Human Protein Atlas. Mutations of the *CSF1R* gene codes are transcribed to mutant CSF1R protein with suboptimal receptor function that leads to dysfunctional monocytic lineage cells with reduced survival and abnormal distribution (93). Thus, impaired microglia are recognized as a primary causative factor underlying *CSF1R*-related leukoencephalopathy.

The strong link between *CSF1R* mutations and pathologic microglia has resulted in further classification of *CSF1R*-related leukoencephalopathy as a CNS primary microgliopathy (91). Replacement of microglia by proliferation of resident microglia, infiltration of microglia-like cells and HSCT (described above) have been evaluated as therapeutic approaches for *CSF1R*-related leukoencephalopathy in mouse models and shown preliminary evidence of efficacy (91). Future human case studies and clinical trials of these microglial replacement therapies will assess the efficacy and safety in patients with ALSP.

The response of microglial cells to changes in the environment of the CNS is activated through a triggering receptor on myeloid cells 2 (TREM2) and its associated protein kinase complex, kDa transmembrane protein (DAP12) (94). The TREM2/DAP12 complex initiates a signaling cascade, including phosphorylation of the spleen tyrosine kinase (SYK). This phosphorylation subsequently activates multiple intracellular pathways of microglia controlled by TREM2 signaling cascades, including phosphatidylinositol 3-kinase, protein kinase C, extracellular regulated kinase, Akt serine/threonine kinase and the elevation of intracellular calcium (95, 96). TREM2-mediated activation pathways include phagocytosis, cell survival and proliferation, modulation of inflammation and regulation of lipid metabolism (97, 98) potentially enabling neuroprotection and nerve tissue regeneration. Mutations in TREM2/DAP12 are associated with the autosomal recessive disorder Nasu-Hakola disease, which is characterized by bone cysts, muscle wasting, and demyelination phenotypes (58). Animal and human genetic studies have demonstrated that microglia without TREM2 or with mutated TREM2 do not convert to an activated stage and subsequently lead to development and/or progression of neurologic disorders. Furthermore, animal models of PD, AD, ALS and demyelinating disease display dysregulation of TREM2/DAP12 signals that promotes pathogenesis of neurodegeneration (94). Based on extensive biochemical research, CSF1R and TREM2 share significant portions of their signaling function that converge via DAP12 and SYK phosphorylation. ALSP is presumably caused by haploinsufficiency of CSF1R due to inactivating mutations, usually in the kinase domain, which results in dysfunctional microglia leading to neurodegenerative pathology, such as neuroinflammation, dementia, and white matter loss (29). Therefore, the signaling pathway convergence between TREM2 and CSF1R, shown in Figure 4, provides the possibility for TREM2 activation to rescue or compensate for CSF1R loss of function (94).

It is apparent that there is dearth of therapies that directly target the etiology of ALSP. Clinical studies should be conducted to develop safe and effective therapies that address the etiology of ALSP, a rare, debilitating and life-threatening neurologic disorder.

## Potential Efficacy Endpoints For Future Interventional Trials

Due to the relatively recent identification of the *CSF1R* gene mutations, specific clinical trial methodologies for ALSP are still evolving. Meaningful biomarkers and efficacy endpoints that capture disease progression will be essential for interventional clinical trials of ALSP. Based upon case studies of ALSP, neuroimaging markers (MRI and CT) and clinician- and patient-rated scales for cognitive, psychiatric and motor dysfunction, coupled with global impression of change assessments, quality of life and disability evaluations, will likely yield convergent endpoints to evaluate efficacy and justify clinical meaningfulness of novel therapeutic agents. Given the similarity of some signs and symptoms of ALSP with FTD, PD and MS, some of the clinical trial methodologies for efficacy endpoints of the related neurologic disorders may be applicable to clinical trials of ALSP.

### Cognitive Decline

Cognitive decline is a clinically significant symptom of ALSP that presents early in the disorder and progresses rapidly (6, 14). In view of this finding, identification of cognitive assessments that capture deficits and decline is an important predictive clinical endpoint for interventional clinical trials of ALSP. Effective scales for measurement of cognitive decline should be fully validated, have inter-relater reliability, show sensitivity to early cognitive changes as well capture decline during progression of the disease and be acceptable to both the patient and assessor (99). Reports of the performance of patients with ALSP on specific cognitive tests have been limited to case reports (63). Given these limitations and the known localization of pathology in patients with ALSP, cognitive tests probing frontal lobe functions, both cortical and subcortical, including attention, processing speed, working memory and cognitive flexibility, are of particular interest.

The Montreal Cognitive Assessment (MoCA) is a validated tool for rapid screening of mild cognitive impairment (100, 101). The MoCA is extensively used as a clinical trial endpoint and for clinical screening in practices to measure cognitive dysfunction in patients with PD or dementia because of its high sensitivity (100%) and specificity (87%) (102, 103).

Other commonly used assessment scales for cognitive decline are the Mini-Mental State Examination (MMSE) (104) and Mini-Cog Test (105). The MMSE has acceptable test-retest and inter-relater reliability, with a sensitivity of 69-91% and a specificity of 87-99% (103). The MMSE has advantages over other scales because of its ability to quantitate cognitive dysfunction over time and to definitively assess treatment effects in clinical trials. A disadvantage of MMSE is its lack of sensibility to mild cognitive disruptions, particularly those related to frontal lobe dysfunction (106).

Several other cognitive tests may be appropriate for ALSP clinical trials. The Trail Making Test (TMT) measures cognition related to processing speed, sequencing, mental flexibility and visual/motor skills (107). The Wisconsin Card Sorting Test (WCST) is aimed at higher level cognitive processes, such as attention, perseverance, abstract thinking and adaption to change (108). The WCST employs two card packs with four stimulus cards and 64 response cards in each pack. The Symbol Digit Modalities Test (SDMT) examines divided attention, visual scanning, tracking, processing speed and motor speed (109). A smartphone-based symbol-digit SDMT has recently been developed to reduce the time of the test (110). The Verbal Fluency Test is primarily designed to measure executive dysfunction (111). The Stroop Color and Word Test (SCWT) assesses the ability to inhibit cognitive interference that takes place when the processing of an initial stimulus affects the simultaneous processing of a second stimulus (112). Due to the reduced capacity of ALSP patients to focus on tasks for a prolonged period of time along with loss of speech and dysfunctional arms and hands, it will be critical to select a battery of cognitive tests that have the flexibility to conduct simple and time expedient verbal or written versions.

Additional clinical scales of cognitive dysfunction that have the potential to yield endpoints for clinical trials of ALSP are the well-known CERAD test battery for AD, Saint Louis University Mental Status (SLUMS) (113), the Memory Impairment Screen (MIS) (114, 115), Clock Drawing Test (CDT) (116), Clinical Dementia Rating (CDR) scale (117), CDR plus National Alzheimer's Coordinating Center Frontotemporal Lobar Degeneration (FTLD) rating (118) and Neuropsychiatry Unit Cognitive Assessment Tool (NUCOG) (119). These scales (described in Supplementary Table 3) are semi-quantitative and have acceptable levels of sensitivity and specificity. Further evaluation of these scales will determine their degree of applicability as endpoints for therapeutic clinical trials of ALSP.

### Motor and Sensory Dysfunctions

Scales that are specifically designed to score motor and sensory dysfunctions of ALSP are not currently available. As the early and progressive stages of ALSP display some motor and non-motor symptoms that are comparable to PD (14), scales used in PD clinical trials may serve as predictive clinical endpoints for interventional clinical trials of ALSP.

Based upon the four components of the Movement Disorder Society (MDS)-sponsored revision (120) of the Unified Parkinson's Disease Rating Scale (MDS-UPDRS), this validated scoring system may be applicable to ALSP. The MDS-UPDRS items are rated by clinicians, patients or both. There are four main components each of which includes several questions related to sensory and motor disabilities. With the exclusion of part 4 which quantifies complications of dopamine replacement therapies, the MDS-UPDRS is a comprehensive scoring tool for motor and sensory complications and quality of life and, therefore, may serve as a clinically meaningful and predictive endpoint to assess therapies in interventional clinical trials of ALSP. However, the overlapping pyramidal and extrapyramidal symptoms in ALSP may introduce scoring limitations and should be considered during the endpoint selection process.

The EDSS is a frequently used disability scale for clinical trials in MS with high relevance to symptoms and functions of patients with ALSP (121). Due to some similarity in the symptoms of ALSP and MS, the EDSS may be another tool to quantify disability endpoints over time in clinical trials of ALSP. The following functional systems are rated: pyramidal (muscle weakness), cerebellar (ataxia, loss of balance, tremor), brainstem (speech problems, swallowing, nystagmus), sensory (numbness, loss of sensation), bowel and bladder (incontinence), visual and cerebral (thinking, memory, fatigue), walking distance and usage of walking aid and/or extension wheelchair.

Several tools that directly measure how a patient functions (gait, strength, spasticity) or feels may provide clinically meaningful endpoints for clinical trials in ALSP. Measurement of the distance walked within a specific time period is evaluated by the 2- or 6-min walk test (122). The Timed Up and Go (TUG) test identifies mobility and balance abnormalities. (123). The Spastic Paraplegia Rating Scale (SPRS) is a reliable and validated measurement of the severity and progression of spasticity (124). Limb and muscle spasticity can also be scored by the Modified Ashworth Scale (MAS), a scale that is widely used in clinical practice for rating the spasticity of extremities and in clinical trials for an assessment of the therapeutic efficacy of spasticity (125).

### Impaired Activities of Daily Living and Physical and Behavioral Dysfunction

Several scales rate the patient's feelings about how daily activities of life are affected by their health disorder. These additional scales may serve as congruent, clinically predictive endpoints of efficacy in clinical trials of therapeutic agents for ALSP. The Goal Attainment Scale (GAS) is a clinician- and patient-scored tool that rates three health goals that are developed by the patient. The patient's progress toward goal achievement is evaluated on a five-point scale from −2 (unfavorable) to +2 (best anticipated outcome) (126). The visual analog scale (VAS) (127), Clinical Global Impressions of Severity (CGI-S) and Improvement (CGI-I) (128), 36-item Short Form Health Survey (SF-36) (129) and Schwab and England Activities of Daily Living (ADL) for PD (130) (described in Supplementary Table 4) are widely accepted global scoring systems for MS and PD and, therefore, may be beneficial for patient and clinician assessment of the dysfunctional effects of ALSP on activities of daily life. Disability and functional scales (described in Supplementary Table 4), such as the Total Functional Capacity (TFC) for Huntington's Disease (131), the Karnofsky Performance Status (KPS) (132) and the Cortical Basal ganglia Functional Scale (CBFS) (133) may also prove to yield clinically meaningful endpoints for ALSP clinical trials. The EuroQOL 5 dimensions questionnaire (EQ-SD) scale is used for MS patients and may be applicable to ALSP (134).

Scales that measure behavioral impairment of patients are likely to generate clinically meaningful data in clinical trials of ALSP (Supplementary Table 5). These widely used neuropsychiatric tools evaluate depression with the Hamilton Rating Scale for Depression (HAM-D) (135, 136) and the Beck Depression Inventory (BDI) (137, 138) and psychosis using the Neuropsychiatric Interview (NPI) (139). Hospital, anxiety, depression scale (HADS) is a useful self-assessment scale for anxiety and depression and a screening tool for mood disorders (140).

Given the severity and devastating progression of ALSP, it will be meaningful to evaluate the behavioral burden that affects the daily lives of caregivers. The Zarit Burden Interview (ZBI) is a commonly used measure of caregiver burden (141). This scoring system covers a wide range of dimensions that include consequences of caregiving, patient dependence, exhaustion and uncertainty, guilt or self-criticism, embarrassment, anger or frustration, psychological burden, emotional reactions and personal/role strain. ZBI has shown acceptable internal consistency and excellent reliability and validity in patients with dementia (142).

Finally, given the heterogeneity of potential symptom presentations and combinations in ALSP, particularly in the early symptomatic stages, patient-specific and patient-centered outcome measures may also be valuable approaches (143).

### Magnetic Resonance Imaging and Other Biomarker Surrogates

Analysis of MRI scans has demonstrated that white matter damage is a congruous finding in most patients with ALSP. White matter abnormalities occur early and are progressive. Given the rapid confluence of patchy or focal T2-weighted hyperintensities and progression of cortical atrophy during the course of ALSP, longitudinal MRI scans on a yearly follow-up basis after diagnosis of ALSP have been recommended to generate a more accurate prognosis of the disorder (14) and may serve as reliable surrogate endpoints for clinical trials.

The MRI severity scoring system, which was developed based on MRI scans of 15 patients with ALSP, may serve as an endpoint for interventional trials of ALSP. The semi-quantitative severity scoring of white matter lesions and brain atrophy is based on a range of 0 (minimal severity) to 57 (maximum severity). Analysis of MRI scans from these 15 patients showed that 14 of the patients (93%) had white matter lesions and the mean total severity score was 16.6 points (range, 10-33.5 points). Although this MRI scoring system shows promise, it requires further validation by prospective longitudinal studies of additional patients and a standard imaging protocol (50, 53).

A widely used scoring method for MRIs of leukodystrophies, the Loes severity score, may also serve as a surrogate clinical endpoint for ALSP clinical trials. This scoring method employs a severity score of 0-34 points for white matter lesions and, with minor modifications, has the capability to detect progression, stabilization and improvement of multiple leukodystrophies (144). Definitive longitudinal MRI studies of progression of white matter abnormalities have not been conducted in patients with ALSP. In one case study of a family in which seven members were diagnosed with HDLS, sequential images in the proband (one MRI at the onset of the disorder and two MRIs during the follow-up period) displayed a progressive, confluent frontal predominant HDLS with symmetrical cortical atrophy (49).

Longitudinal MRI alterations were also detected in seven patients with HDLS through the MRI rating scale. Total MRI scores varied from 12 to 44, white matter lesion scores ranged from 11 to 32 and atrophy scores ranged from 1 to 12. The severity of MRI scores increased significantly with the duration of HDLS (total score, *p* < 0.01, white matter lesion score, *p* < 0.01 and atrophy score, *p* < 0.01). The mean changes in scores on a year basis were 3.7 ± 1.5 for the total score, 2.5 ± 1.1 for white matter lesion score and 1.3 ± 0.5 for the atrophy score (52).

Rapid Estimation of Myelin for Diagnostic Imaging provides a validated and robust myelin quantification that detects diffuse demyelination in normal-appearing tissue in MS (145). This demyelination is associated with both cognitive and clinical disability. Because the technique is rapid with automatic postprocessing and U.S. FDA approval, it may be a clinically feasible biomarker suitable for monitoring myelin dynamics and evaluation of treatments aimed at remyelination of ALSP.

Biochemical assays of levels of various soluble biomarkers in CSF (tau proteins, NfL proteins and glial fibrillary acidic protein) and in plasma (NfL) of ALSP patients may serve as meaningful surrogate endpoints for clinical trials (14, 20, 24, 40, 67, 86). These biomarkers are etiologic factors that underlie neural, axonal and glial cell damage, and may be considered as trial endpoints in conjunction with MRI analysis. Further development and validation of such assays are important for early translational trials in ALSP.

### Digital Biomarkers

Traditional methods of assessing neuromotor disorders, such as clinical rating scales, are subjective and prone to human bias. During the last decade, a multitude of technology-based objective measures of human behavior and function have been developed, bringing with them the promise of substantial change to the diagnostic, monitoring and therapeutic landscape in neurodegenerative diseases (146, 147). Sensors, mobile communications, cloud computing, advanced analytics and the Internet of Things (wireless connectivity of all electronic devices) are among the innovations that have the potential to transform healthcare and the approach to patients with chronic, complex and fluctuating disorders (148). These devices offer potential novel approaches to more accurately assess motor dysfunction in interventional clinical trials of ALSP.

Wearable activity trackers are electronic monitoring devices that enable users to track and monitor their health-related physical fitness metrics, including the number of steps taken, level of activity, walking distance, heart rate and sleep patterns. Despite the proliferation of these devices in various contexts of use and rising research interest, there is limited understanding of the broad research landscape (149).

Although commercial grade activity monitors like Fitbits and the Apple Watch provide objective data, the results are limited to activity tracking only. Medical-grade wearable precision motion sensor solutions overcome these limitations. Such platforms can deliver objective, high-frequency data combined with scientifically validated endpoints that are specific to a patient population (148).

The use of consumer wearable technologies in medicine is becoming increasingly more common. For instance, in the field of sleep medicine, the use of actigraphy for sleep monitoring may be used to supplant more traditional methods like polysomnography due to its validity, lower cost and ability to evaluate individuals in their homes over a longer period of time (150). Advanced wearable technologies can also precisely monitor skin conductance, respiratory rate, blood pressure and oximetry and provide surface electromyography (EMG), electrocardiography (ECG) and electroencephalograpy (EEG) tracings. Furthermore, the ability to collect multiple aspects of human function with smart devices (mobile phones, tablets and smart watches) provides additional opportunities to collect and analyze numerous clinically relevant parameters (e.g., posture, balance, gait, dexterity, voice and speech patterns, facial expression, eye tracking, medication).

Development of precision medicine subtypes for common diseases, such as PD and AD, and deep phenotype maps based on digital sensing technologies of rare disease populations, such as ALSP, could capture therapeutic responsiveness to experimental treatment paradigms (151–156).

## Patient and Caregiver Perspective of Burden Due to Unmet Medical Need

Progressive neurologic disorders invoke a heavy burden on afflicted patients, caregivers and society (157). Although data on the patient and caregiver burden of ALSP are unavailable, data from other progressive neurological disorders provide insight into the potential burden of ALSP on patients and caregivers.

Research questionnaires have been used to understand the unmet medical needs of progressive neurologic disorders by directly seeking input from the afflicted patients. A cross-sectional study of 1,205 patients with MS was conducted using a questionnaire to collect information on demographics (sex, age, education, employment), clinical status (form, disease duration, disability level) and unmet healthcare and social needs (12 items scored as yes/no) (158). Psychological support (27.5%) was the greatest unmet healthcare need, followed by temporary admission to rehabilitation (9.8%), access to technical aid (6.7%), access to drugs (5.4%) and lack of nursing home admission (3.2%). Social care needs included assistance with transportation (41%), financial support, architectural barriers, personal assistance (>30%), career guidance and adaptation to workplace (>10%). Unmet healthcare needs were linked to clinical factors, such as disease progression and level of disability.

Because there is a paucity of data underlying the major burden of the physical, psychological, emotional and financial impositions of ALSP on patients, families and caregivers, it is appropriate to examine the burden of closely related neurologic disorders, such as ALS and FTD. ALS and FTD are both characterized as rare diseases by Genetic and Rare Diseases (GARD), National Organization for Rare Disease (NORD) and Orphanet. These are meaningful comparative disorders for ALSP because both are rare diseases that affect motor function, cognition and mental health and have unmet medical needs.

The burden of devastating symptoms of ALS disrupts quality of life and shortens the lifespan of patients. The financial burden to patients, families and payers is substantial. A case study of the costs of care for individual patients was conducted in the United States over a 10-year period (2001-2010) (159). Total costs for the duration of ALS were $1,433,992 (85% paid by insurance, 9% paid by patient and family and 6% paid by charity). The greatest costs involved in-home caregivers ($669,150), ventilation ($212,430) and hospital care ($114,558). These cost factors are particularly burdensome for patients because they markedly influence treatment decisions. It is important to note that this case study did not address the indirect financial detriments to patients, such as lost wages, productivity and terminated employment.

The burden of the progressive symptoms of FTD and the economic burden are staggering for patients, families and caregivers. A robust 250-item survey was administered to primary caregivers of patients with FTD to estimate the cost burden of the disorder (160). The survey was completed by 674 of the 956 caregivers. Direct and indirect annual costs of FTD were $47,916 and $71,737, respectively, and resulted in an annual per-patient charge of $119,653. Elderly patients with later stages of behavioral-variant FTD had higher direct costs whereas male patients aged <65 years had higher indirect costs. The impact of FTD on the patient and family resulted in a mean reduction in household income ranging from $75,000 to $99,000 (12 months before diagnosis) to $50,000 to $59,000 (12 months after diagnosis). This dramatic loss of household income was related to lost days of employment and to early departure from employment. The profound economic burden of FTD may be reduced in the future through accurate and early diagnosis, effective treatments to target cause of the disorder and improved professional services.

The financial burden of MS has been explored in cohort studies conducted with patient data extracted from the Swedish Multiple Sclerosis Register (SMSreg). These MS studies have shown that the level of cognitive function correlates directly with the amount of work disability (161) and quantity of income independent of physical disability (162). Patients with relapsing, remitting MS also earned twice the income of patients with progressive MS (163).

ALSP is a rare, progressive, debilitating disorder and its treatment is an unmet medical need. The signs and symptoms of ALSP present a major burden for daily living, cost of care and life expectancy of afflicted patients. The treatment of ALSP will require a patient-focused, precision medicine therapeutic approach by the multidisciplinary caregiver team and foundation and support groups to address the cause of the disorder, management of motor and sensory symptoms and careful attention to quality of life issues.

## Gap Analysis of Clinical Manifestations

There is a paucity of published clinical research literature for ALSP, a rare neurodegenerative disorder. The limited number of published clinical research studies is comprised primarily of case reports with small numbers of patients and absence of controls. Therefore, formal gap analysis of ALSP clinical manifestations was not conducted for this comprehensive review. In an effort to gain some understanding of the gaps in clinical manifestations of ALSP, a count of ALSP-specific publications was conducted. Table 1 lists the references and total number of references for publications that are ALSP-specific for each of the clinical manifestations. The most conspicuous gaps in the literature were identified as the potential efficacy endpoints for future clinical trials. Endpoints such as cognitive decline, motor and sensory dysfunction, impaired activities of daily living with physical and behavioral dysfunction, digital biomarkers and patient and caregiver perspective of burden due to unmet medical need had the fewest (0-3) ALSP-specific literature references. These gaps are likely related to the low global incidence of ALSP patients which have restricted the number of adequate and controlled clinical trials. Future clinical studies of ALSP should target the development of clinically meaningful, congruent, specific and validated efficacy endpoints that will accelerate the discovery of safe and effective therapies for this rare disorder.

**Table 1:** Gaps in ALSP specific published literature of clinical manifestations.

**Clinical manifestations of ALSP**	**ALSP-specific literature references**	**Total ALSP-specific literature references**
Genetics	(6, 9, 13, 14, 17–42)	(30)
Neuropathology	(6, 14, 17, 28, 29, 32, 43–48)	(12)
Imaging	(1, 6, 13, 22, 47, 49–59)	(16)
Prevalence	(5, 6, 9, 13, 14, 58, 60, 61)	(8)
Clinical course	(6, 13, 14, 53, 62, 63)	(6)
Diagnosis and clinical evaluation	(13, 14, 30, 31, 53, 64–67)	(9)
Current treatment for management of symptoms	(6, 14, 27, 44, 48, 68–72)	(10)
Potential neuropathophysiologic biomarkers	(28, 33, 62, 67)	(4)
Current and proposed interventional clinical studies of therapeutics	(27, 29, 48, 58, 70, 71, 91, 94)	(8)
Cognitive decline endpoint	(6, 14, 63)	(3)
Motor and sensory dysfunction endpoints	(14)	(1)
Impaired activities of daily living and physical and behavioral dysfunction endpoints	None	None
Magnetic resonance imaging and other biomarker surrogate endpoints	(14, 20, 24, 49, 50, 52, 53, 67)	(8)
Digital biomarker endpoints	None	None
Patient and caregiver perspective of burden due to unmet medical need	None	None

## Limitations

There were limitations to this comprehensive review of the clinical manifestations of ALSP. Most of the clinical data were derived from limited numbers of patients in published case studies. Due to the paucity of ALSP-specific clinical literature, some gaps were evident in the clinical manifestations of the disorder, particularly efficacy endpoints. Lack of patient medical records associated with the case studies may have resulted in inaccurate, incomplete or missing assessments of symptoms and disease progression. Quality control of case studies was restricted to inclusion and exclusion criteria with no additional quality parameters. There was considerable variation in geographic location of the clinics involved in the case studies and this may have created inconsistent interpretation of the clinical manifestations.

## Conclusions

This comprehensive clinical review of the literature focused on the genetics, neuropathology, imaging findings, prevalence, clinical course, diagnosis and clinical evaluation of ALSP, as well as on prospective biomarkers, current and proposed treatment, promising clinical scales and efficacy endpoints for future therapeutic trials and the burden of ALSP on patients and caregivers. The description of the clinical manifestations of ALSP was derived primarily from clinical case studies with small numbers of patients. Due to the paucity of non-interventional and interventional clinical studies of ALSP, the information gained from this review can serve as a foundation for the strategy and design of future clinical trials, with clinically meaningful and congruent efficacy endpoints for patients with ALSP. These clinical trials will be designed to elicit determinative assessments for the development of therapeutics for ALSP, an orphan neurodegenerative disease with an unmet medical need, target with precision the etiology and alleviate symptoms in an effort to reverse, halt or slow progression of ALSP.

## Author Contributions

SP, AP, MB, and SZ were involved in the strategy, conception of work, literature search, and writing and revision of manuscript. EF, VK, DL, WK, LS, SH, TK, TI, TL, JO-M, FE, and ZKW read and critically revised the manuscript. All authors approved the final manuscript for submission.

## Conflict of Interest

Unrelated to this study, EF received personal compensation for serving on a PSP Scientific Advisory or Data Safety Monitoring board for Biogen, Vigil Neuroscience, Inc., and Denali Therapeutics, as a section editor for NeuroImage Clinical and as a course director for the AAN Annual Meeting. EF has received research support paid to her institution (UWO) from CIHR and the Weston Foundation to conduct an ongoing study of oxytocin in FTD, from Alzheimer Society of Canada and the Physicians and Services Incorporated Foundation, the Ministry of Research and Innovation of Ontario for research and for site participation in clinical trials sponsored by Alector, Biogen, and TauRx. VK was funded by the Stockholm County Council. WK received consulting honoraria from Vigil Neuroscience. LS was funded by the German Research council (DFG grant SCHO754/6-2), German Ministryof Health (BMG grant ZMVI1-2520DAT94E to LeukoExpert), German Ministry of Education and Research (BMBF grant 01GM1905A to Treat HSP and grant 01GM1907A to Treat ION), European Commission (EU grant 947588 to the ERNRND registry and JPND grant 01ED16028 to ESMI). LS was a member of the European Reference Network for Rare Neurological Diseases (Project No 739510). SH was funded by the Hertie Network of Excellence in Clinical Neuroscience (GHST grant P1200021). TK and TI are funded by AMED JP21dk0207045, a public grant from the Japanese government to support research on ALSP. JO-M was funded by the Conrad N. Hilton Foundation, the Institute for Translational Medicine and Therapeutics Transdisciplinary (ITMAT) and serves as a principal investigator on Vigil Neuroscience, Inc. sponsored clinical studies (VGL101-01.001; VGL101-01.002). FE is the principal investigator of Bluebird Bio and Minoryx Therapeutics clinical trials; consultant to Ionis, Alnylam, Sanofi Genzyme, Minoryx, and SwanBio Therapeutics; director of the Third Rock MGH Neuroscience Fellowship; and founder of SwanBio Therapeutics. ZW was partially supported by the NIH/NIA and NIH/NINDS (1U19AG063911, FAIN: U19AG063911), Mayo Clinic Center for Regenerative Medicine, Mayo Clinic in Florida Focused Research Team Program, gifts from the Sol Goldman Charitable Trust and Donald G. and Jodi P. Heeringa Family, the Haworth Family Professorship in Neurodegenerative Diseases fund, and the Albertson Parkinson's Research Foundation. He serves as PI or Co-PI on Biohaven Pharmaceuticals, Inc. (BHV4157-206 and BHV3241-301), Neuraly, Inc. (NLY01-PD-1), and Vigil Neuroscience, Inc. (VGL101-01.001) clinical studies. He serves as an external advisory board member for Vigil Neuroscience, Inc. SP, AP, MB, and SZ are employed by Vigil Neuroscience, Inc. The remaining authors declare that the research was conducted in the absence of any commercial or financial relationships that could be construed as a potential conflict of interest.

## Publisher's Note

All claims expressed in this article are solely those of the authors and do not necessarily represent those of their affiliated organizations, or those of the publisher, the editors and the reviewers. Any product that may be evaluated in this article, or claim that may be made by its manufacturer, is not guaranteed or endorsed by the publisher.
