# Supplementary material for: Adult-Onset Leukoencephalopathy With Axonal Spheroids and Pigmented Glia: Review of Clinical Manifestations as Foundations for Therapeutic Development
Source: Front Neurol. 2022 Feb 3;12:788168. doi: 10.3389/fneur.2021.788168 (PMC8850408; doi:10.3389/fneur.2021.788168)
Supplement: Supplementary file 1 [file Table_1.docx]

**SUPPLEMENTARY MATERIAL**

**Table 1. Genetic Mutations in CSF1R-related Leukoencephalopathy**

| **Location** | **Nucleotide Change** | **Amino Acid Change** | **Protein Region** |
| --- | --- | --- | --- |
| Exon 2 | c.49G > A | p.Gly17Cys | Signal peptide |
| Exon 2 | c.49G > T | p.Gly17Val | Signal peptide |
| Exon 4 | c.310delC | p.Pro104Leufs*8 | Ig |
| Exon 8 | c.1085A > G | His362Arg | Ig |
| Exon 11 | - | p.Tyr540* | Transmembrane Domain |
| Exon 12 | c.1699delA | p.Thr567fs*44 | Juxtamembrane Domain |
| Exon 12 | c.1745T > C | p.Leu582Pro | TKD |
| Intron 12 | c.1754-2A > G | p.Gly585_Lys619delinsAla | TKD |
| Exon 13 | c.1765G > A | p.Gly589Arg | TKD |
| Exon 13 | c.1766G > A | p.Gly589Glu | TKD |
| Exon 13 | c.1786G > A | p.Val596Met | TKD |
| Intron 13 | c1858+1G > T | Exon 13 skipped | TKD |
| Exon 14 | c.1889T > G | p.Leu630Arg | TKD |
| Exon 14 | c.1897G > A | p.Glu633Lys | TKD |
| Exon 14 | c.1901T > G | p.Leu634Arg | TKD |
| Exon 14 | c.1907T > A | p.Ile636Asn | TKD |
| Exon 14 | c.1952G > A | p.Gly651Glu | TKD |
| Exon 14 | c.1954G > C | p.Ala652Pro | TKD |
| Exon 14 | c.1957T > C | p.Cys653Arg | TKD |
| Exon 14 | c.1958G > A | p.Cys653Tyr | TKD |
| Exon 14 | C1985T > C | p.Ile662Thr | TKD |
| Exon 14 | c.1987G > A | p.Glu663Lys | TKD |
| Exon 15 | c.1990G > A | p.Glu664Lys | TKD |
| Exon 15 | c.1996C > T | p.Cys666Arg | TKD |
| Exon 15 | c.2026C > T | p.Arg676* | KID |
| Exon 15 | c.2060_2061insT | p.Ser688Glu fs*13 | KID |
| Exon 17 | - | p.Phe758Ser | TKD |
| Exon 17 | c.2276C > T | p.Ser759Phe | TKD |
| Exon 17 | c.2287G > C | p.Ala763Pro | TKD |

| **Location** | **Nucleotide Change** | **Amino Acid Change** | **Protein Region** |
| --- | --- | --- | --- |
| Exon 17 | c.2287G > A | p.Ala763Pro | TKD |
| Exon 17 | c.2294G > A | p.Gly765Asp | TKD |
| Exon 17 | c.2296A > G | p.Met766Val | TKD |
| Exon 17 | c.2297T > C | p.Met766Thr | TKD |
| Exon 17 | c.2308G > C | p.Ala770Pro | TKD |
| Intron 16 | c.2319+1C > A | Not Characterized | -- |
| Intron 17 | c.2320-2A > G | p.Cys774_Asn814del | TKD |
| Exon 18 | c.2324T > A | p.Ile775Asn | TKD |
| Exon 18 | c.2329C > T | p.Arg777Trp | TKD |
| Exon 18 | c.2330G > A | p.Arg777Gln | TKD |
| Exon 18 | c.2334C > A | p.Asp778Glu | TKD |
| Exon 18 | c.2342C > T | p.Ala781Val | TKD |
| Exon 18 | c.2342C > A | p.Ala781Glu | TKD |
| Exon 18 | - | p.Arg782Cys | TKD |
| Exon 18 | c.2344C > G | p.Arg782Gly | TKD |
| Exon 18 | c.2345G > A | p.Arg782His | TKD |
| Exon 18 | c.2350G > A | p.Val784Met | TKD |
| Exon 18 | c.2375C > A | p.Ala792Asp | TKD |
| Exon 18 | c.2378A > C | p.Lys793Thr | TKD |
| Exon 18 | c.2381T > A | p.Ile794Phe | TKD |
| Exon 18 | c.2381T > C | p.Ile794Thr | TKD |
| Intron 18 | c.2442+2T > C | N/A | -- |
| Intron 18 | c.2442+1G > A | N/A | -- |
| Intron 18 | c.2442+1G > T | N/A | -- |
| Intron 18 | c.2442+5G > A | N/A | -- |
| Intron 18 | c.2442+5G > C | p.Cys774_Asn814delinsGlnGlyLeuGlnSerHisVa l GlyProSerLeuProSerSerSerProGlnAlaGln | TKD |
| Exon 19 | c.2450T > A | p.Leu817Gln | TKD |
| Exon 19 | c.2450T > C | p.Leu817Pro | TKD |
| Exon 19 | c.2467C > T | p.Ala823Val | TKD |
| Exon 19 | c.2468C > A | p.Ala823Asp | TKD |
| Exon 19 | c.2470C > T | p.Pro824Ser | TKD |
| Exon 19 | c.2473G > A | p.Glu825Lys | TKD |
| Exon 19 | c.2480T > C | p.Ile827Thr | TKD |

| **Location** | **Nucleotide Change** | **Amino Acid Change** | **Protein Region** |
| --- | --- | --- | --- |
| Exon 19 | c.2483T > C | p.Phe828Ser | TKD |
| Exon 19 | c.2498C > A | p.Thr833Lys | TKD |
| Exon 19 | c.2509G > T | p.Asp837Tyr | TKD |
| Exon 19 | c.2512G > C | p.Val838Leu | TKD |
| Exon 19 | c.2522A > G | p.Tyr841Cys | TKD |
| Exon 19 | c.2525G > T | p.Gly842Val | TKD |
| Exon 19 | c.2527A > T | p.Ile843Phe | TKD |
| Exon 19 | c.2527_2530delins GGCA | p.Ile843_Leu844delinsGlyIle | TKD |
| Exon 19 | c.2528T > A | p.Ile843Asn | TKD |
| Exon 19 | c.2534T > C | p.Leu845Pro | TKD |
| Exon 19 | c.2539G > A | p.Glu847Lys | TKD |
| Exon 19 | c.2540A > T | p.Glu847Val | TKD |
| Exon 19 | c.2541G > C | p.Glu847Asp | TKD |
| Exon 19 | c.2546_2548delTCT | p.Phe849del | TKD |
| Exon 19 | c.2546T > C | p.Phe849Ser | TKD |
| Exon 19 | c.2552T > C | p.Leu851Pro | TKD |
| Exon 20 | c.2562T > A | p.Asn854Lys | TKD |
| Exon 20 | c.2563C > A | p.Pro855Thr | TKD |
| Exon 20 | c.2566T > C | p.Tyr856His | TKD |
| Exon 20 | c.2570C > T | p.Pro857Leu | TKD |
| Exon 20 | c.2603T > C | p.Leu868Pro | TKD |
| Exon 20 | - | p.Val869Gly | TKD |
| Exon 20 | c.2624T > C | p.Met875Thr | TKD |
| Exon 20 | c.2629C > T | p.Gln877* | TKD |
| Exon 20 | c.2632C > G | p.Pro878Ala | TKD |
| Exon 20 | c.2632C > A | p.Pro878Thr | TKD |
| Exon 20 | c.2632C > T | p.Pro878Ser | TKD |
| Exon 20 | c.2645delC | p.Pro882Pro fs*70 | TKD |
| Intron 20 | c.2655-2A > G | N/A | -- |
| Exon 21 | -- | p.Val886Gln fs*55 | TKD |
| Exon 21 | -- | p.Cys892_Ala894del | TKD |
| Exon 21 | c.2699G > A | p.Arg900Lys | TKD |
| Exon 21 | c.2701C > T | p.Pro901Ser | TKD |

| Exon 21 | c.2717 T > C | p.Ile906Thr | TKD |
| --- | --- | --- | --- |
| Exon 22 | -- | p.Phe970Ser fs*108 | C terminus |
| Exon 22 | -- | p.Phe970Ser fs*7 | C terminus |

Abbreviations: Ig = immunoglobulin, KID = kinase inhibitory domain, N/A = not applicable, TKD = tyrosine kinase domain

Genetic mutations in this table were extracted from Stanley ER, Chitu V. CSF-1 receptor signaling in myeloid cells. *Cold Spring Harbor Perspect Biol.* (2014) Jun2;6(6): a021857. doi: 10.1101/cshperspect.a021857.

**Table 2. Diagnostic Criteria for ALSP**

| **Core features**   1. Age at onset ≤ 60 years 2. More than 2 findings of the following clinical signs and symptoms    1. Cognitive impairment or psychiatric symptoms    2. Pyramidal signs    3. Parkinsonism    4. Epilepsy 3. Autosomal dominant inheritance or sporadic occurrence 4. Brain CT/MRI findings    1. Bilateral cerebral white matter lesions    2. Thinning of the corpus callosum 5. Other causes of leukoencephalopathy including vascular dementia, multiple sclerosis, or leukodystrophy (e.g. adrenoleukodystrophy, Krabbe disease, metachromatic leukodystrophy) can be excluded   **Exclusionary findings**   1. Age at onset ≤ 10 years 2. Stroke-like episodes more than twice except for epilepsy 3. Prominent peripheral neuropathy   **Supportive findings**   1. Frontal lobe dysfunction shown by clinical features or cognitive battery test 2. Rapidly progressive course. Become bedridden within 5 years after onset 3. Spotty small calcifications in the white matter shown by brain CT 4. Neuropathologic findings compatible to ALSP |  |
| --- | --- |
|  | |
| **Diagnosis by the criteria** |  |
| **Definite:** Fulfill core features 2, 3, and 4a and confirmation of *CSF1R* mutation |  |
| **Probable:** Fulfill core features 1–5, but genetic tests have not been performed |  |
| **Possible:** Fulfill core features 2a, 3, and 4a, but genetic tests have not been performed |  |

ALSP, Adult-onset leukoencephalopathy with axonal spheroids and pigmented glia; CSF1R, colony stimulating factor 1 receptor; CT, computed tomography; MRI, magnetic resonance imaging

Reprinted with permission from Konno T, Yoshida K, Mizuta I, Mizuno T, Kawarai T, Tada M, et al. Diagnostic criteria for adult-onset leukoencephalopathy with axonal spheroids and pigmented glia due to CSF1R mutation. *Eur J Neurol*. (2018) Jan;25(1):142-7. doi: 10.1111/ene.1346

**Table 3. Additional Clinical Scales of Cognitive Dysfunction**

| **Scale** | **Type** | **Overview of Scoring System** |
| --- | --- | --- |
| CERAD Test Battery | Clinician rated | Test battery for screening and diagnosis of cognitive deficits in Alzheimer’s disease patients and consists of 7 subtests (MMSE, verbal fluency, MBNT, construction ability, learning of word lists for recall and recognition, vocabulary test and visuo-motor tracking) |
| St. Louis University Mental Status (SLUMS) | Clinician rated | 7-minute, 11-item questionnaire with scores from 0 to 30 that measure orientation, memory, attention, and executive functions related to dementia and neurocognitive disorder |
| Memory Impairment Screen (MIS) | Clinician rated | 4-minute, 4-item delayed free and cued recall test of memory impairment for Alzheimer’s disease and other dementias |
| Clock Drawing Test (CDT) | Clinician rated | 3-minute semiquantitative or quantitative scoring of patient drawing a clock to measure several cognitive disabilities related to attention, auditory comprehension, verbal working memory, numerical knowledge, visual memory, visuospatial skills, praxis, and executive function associated with several neurodegenerative disorders |
| Clinical Dementia Rating (CDR) Scale | Clinician rated | Assessment of cognitive and functional performance in dementia patients via memory, orientation, judgement, and problem solving, community affairs, home and hobbies and personal care with scores ranging from 0 none) to 3 (severe) |
| CDR + National Alzheimer’s Coordinating Center (NACC) Frontotemporal Lobar Degeneration (FTLD) Rating | Clinician rated | Assessment of cognitive and functional performance of the 6 domains of the CDR plus 2 FTLD domains of behavior and language, with scores ranging from 0 (none) to 3 (severe) in dementia patients |
| Neuropsychiatry Unit Cognitive Assessment Tool (NUCOG) | Clinician rated | Cognitive status assessment with pen and paper screening tool of 5 domains (attention, memory, language, executive function, and visuospatial function); scores range from 0 to 20 for each domain in patients with dementia (cut-off score of 80 for dementia) or other neurological disorders |

**Table 4. Additional Clinical Scales of Impaired Activities of Daily Living and Physical Dysfunction**

| **Scale** | **Type** | **Overview of Scoring System** |
| --- | --- | --- |
| Visual Analog Scale (VAS) | Patient rated | Assessment of 15 statements related to fatigue, pain, anxiety, and quality of life, with a score measured on a 10-cm line from 0 mm (none) to 100 mm (severe) in multiple sclerosis (MS)and other neurologic disorders |
| Clinical Global Impressions (CGI) Scale for Severity and Improvement | Clinician rated | Measurement of severity of symptoms, behavior, and function on a scale of 1 (normal) to 7 (extremely ill) and improvement compared to baseline on a scale of 1 (very much improved since treatment) to 7 (very much worse since treatment) in several neurologic disorders |
| 36-Item Short Form Health Survey (SF-36) | Clinician rated | Assessment of 36 items related to 8 scales of physical function, role limitations, body pain, general health, vitality, social function, role limitations, and mental health, with scores ranging from 0 to 100 and lower scores indicative of more disability in Parkinson’s disease and multiple sclerosis |
| Schwab and England Activities of Daily Living (ADL) Scale | Clinician or Patient rated | Measurement of capabilities associated with impaired mobility, with each item scored as a percentage from 0% (vegetative functions, bedridden) to 100% (completely independent) in Parkinson’s disease |
| Total Functional Capacity (TFC) Scale | Clinician rated | Evaluation of self-care (25 yes/no questions), mobility (5 items with 4-point ordinal scale from 0 to 3 with higher values showing better function), and independence (scores from 10 to 100 with higher scores indicating more independence) in Huntington’s disease |
| Karnofsky Performance Status (KPS) | Clinician rated | Scores functional (performance) status on a comprehensive 11-point scale that correlates with percentage values ranging from 100% (no disease or symptoms) to 0% (death) |
| Cortical Basal Ganglia Functional Scale (CBFS) | Clinician and  Patient rated | Scores functional (performance) on 14 items of motor experience and 17 items of non-motor experiences in daily living using a multi-stage Delphi process |
| EuroQOL 5 Dimensions Questionnaire (EQ-5D) | Clinician and  Patient rated | Scores 5 quality of life items (mobility, self-care, usual activities, pain/discomfort and anxiety/depression) with Visual Analog Scale from 0 (worst) to 100 (highest) |

**Table 5. Clinical Scales of Behavioral Dysfunction**

| **Scale** | **Type** | **Overview of Scoring System** |
| --- | --- | --- |
| Hamilton Depression Rating Scale (HAM-D) | Clinician rated | Assessment of 17 to 29 items (depending on version) with a 3- or 5-point Likert scale rating severity of depression (total score of 0-7 not depressed, > 23 very severe depression) based on questions encompassing mood, feelings of guilt, suicide ideation, insomnia, agitation, anxiety, weight loss, and somatic symptoms |
| Beck Depression Inventory (BDI) | Clinician or  Patient rated | Evaluation of 21 items of depression symptoms, including mood, pessimism, sense of failure,  self-dissatisfaction, guilt, punishment, self-dislike, self-accusation, suicidal ideation, crying, irritability, social withdrawal, indecisiveness, body image change, work difficulty, insomnia, fatigability, loss of appetite, weight loss, somatic preoccupation, and loss of libido, with each item rated from 0 to 3 with total scores of 0 to 9 corresponding to no or minimal depression and scores of 30 to 63 corresponding to severe depression |
| Neuropsychiatric Inventory (NPI) row | Clinician rated | Scoring of 12 symptoms (delusions, hallucinations, agitation, depression, anxiety, euphoria, apathy, disinhibition, irritability, aberrant motor behavior, nighttime behavior disturbances, and appetite and eating abnormalities) yielding a total score of 144 with scores <20 corresponding to mild problem and scores >50 corresponding to severe problem for multiple neurological conditions |
| Hospital Anxiety and Depression Scale | Clinician and  Patient rated | Scoring of 7 items for major depression and generalized anxiety disorder from 0 (lowest) to 3 (highest level of depression or anxiety) |
